# Life Modifications and PCOS: Old Story But New Tales

**DOI:** 10.3389/fendo.2022.808898

**Published:** 2022-04-13

**Authors:** Yuanyuan Gu, Guannan Zhou, Fangyue Zhou, Qiongwei Wu, Chengbin Ma, Yi Zhang, Jingxin Ding, Keqin Hua

**Affiliations:** ^1^Changning Maternity and Infant Health Hospital, East China Normal University, Shanghai, China; ^2^Department of Gynecology, The Obstetrics and Gynecology Hospital of Fudan University, Shanghai, China; ^3^Department of Gynecology, Shanghai Key Laboratory of Female Reproductive Endocrine Related Diseases, Shanghai, China; ^4^The International Peace Maternity and Child Health Hospital, School of Medicine, Shanghai Jiao Tong University, Shanghai, China

**Keywords:** polycystic ovarian syndrome (PCOS), lifestyle modifications, insulin resistance, hyperandrogenism, obesity

## Abstract

Polycystic ovary syndrome (PCOS) is defined as a kind of endocrine and metabolic disorder that affects female individuals of reproductive age. Lifestyle modifications, including diet modifications, exercise, and behavioral modification, appear to alleviate the metabolic dysfunction and improve the reproductive disorders of PCOS patients (particularly in obese women). Therefore, lifestyle modifications have been gradually acknowledged as the first-line management for PCOS, especially in obese patients with PCOS. However, the mechanism of lifestyle modifications in PCOS, the appropriate composition of diet modifications, and the applicable type of exercise modifications for specific female populations are rarely reported. We conducted a systematic review and enrolled 10 randomized controlled trials for inclusion in a certain selection. In this review, we summarized the existing research on lifestyle modifications in PCOS. We aimed to illustrate the relationship between lifestyle modifications and PCOS (referring to hyperandrogenism, insulin resistance as well as obesity) and also considered the priorities for future research. These results might be an invaluable tool to serve as a guide in lifestyle modifications as the intervention for PCOS and other related endocrine disorders.

## Background

Polycystic ovary syndrome (PCOS) is a kind of common endocrine and metabolic disorder which disturbs reproductive-age female individuals (1). The symptoms of PCOS involved serious reproductive dysfunctions (including infertility and pregnancy complications) (2, 3), unbalanced metabolic functions (including insulin resistance, type 2 diabetes, and so on) as well as psychological disorders (mainly including depression and anxiety) and other implications (4), which disturb female patients whose ages range from adolescence to menopause. Recently, lifestyle modification is widely considered to be the cornerstone of many endocrine and metabolic disorders (5, 6). An increasing number of studies investigated the effectiveness and the frequency of lifestyle modification management in PCOS treatment (5). It is acknowledged that lifestyle plays a vital role in the development of PCOS, and lifestyle interventions are necessary. It seems that the lifestyle modification for PCOS is an old story without any new tales; however, the kind of lifestyle modification that would be effective for PCOS, the difference between various modifications for different populations, and the underlying mechanism of the modifications are still uncertain. Herein we provide a comprehensive review of the influence of lifestyle modifications on the course of PCOS.

## Methods

### Search Strategy and Design

We selected PubMed database for the systematic literature search. The search strategy included (i) “polycystic ovary syndrome” and its synonyms; (ii) “lifestyle”, “exercise therapy”, “behavior therapy”, “diet therapy”, and their synonyms; and (iii) “weight loss” and its synonyms. The outcome of the literature review was weight loss with different interventions (diverse lifestyle modifications).

### Selection Criteria

We observed the inclusion criteria including (i) randomized controlled trials (RCTs), (ii) participants with polycystic ovary syndrome according to the Rotterdam criteria, (iii) intervention: lifestyle modification (with a clear description of methods) without pharmacological components, (iv) weight loss as outcome, and (v) follow-up of more than 24 weeks.

## Results

From the results in **Table 1**, a healthy eating diet and educational programs would lead to more weight loss. The outcomes after a diet modification, an exercise modification, and a combination of diet and exercise are significantly different. A high-protein diet also leads to weight loss when compared with a standard protein diet. However, there was no significant difference between a vegan diet and a caloric diet. No significant difference was also found between a low-glycemic-index (GI) diet and a hypocaloric healthy eating diet.

**Table 1:** Results of the 10 enrolled randomized controlled trial studies.

Year	Author	Grouping factors	Results				Significance
			Grouping 1	Grouping 2	Grouping 3	Control group	
2018	Oberg E (7)	Behavioral intervention *vs*. minimal intervention	Mean weight loss -2.1%			Mean weight loss -1.0%	*P* = 0.002
2015	Wong J (8)	Low-glycemic-load diet *vs*. low-fat diet	Mean weight loss -1.2 ± 0.8 kg (-1.2%)	Mean weight loss -4.8 ± 1.6 kg (-5.5%)			*P* = 0.02
2015	Marzouk T (9)	Hypocaloric and healthy eating diet + educational program *vs*. healthy eating, no caloric restriction	Mean weight loss 90.8–83.7 kg (-3.9%)	Mean weight loss 90.5–90.1 kg (-0.4%)			*P* = 0.041
2014	Turner G (10)	Vegan diet *vs*. low-calorie diet	Mean weight loss 1.1%	Mean weight loss -0%			Not significant
2012	Sorensen L (11)	High-protein diet *vs*. standard protein diet	Mean weight loss 81.8–74.1 kg (7.7 kg) -9.4%			Mean weight loss 78.7–75.4 kg (3.3 kg) -4.2%	*P* = 0.002
2011	Egan N (12)	Low-glycemic-index diet *vs*. hypocaloric healthy eating diet	Median weight change -5.8 kg	Median weight change -4.5 kg			Not significant
2011	Nybacka A (13)	Diet *vs*. exercise *vs*. diet + exercise	Mean weight loss -6%	Mean weight loss -3%	Mean weight loss -5%		*P* < 0.001
2010	Marsh (14)	Low-glycemic-index diet *vs*. conventional healthy diet	Mean weight loss -5.2%, ITT: -3.2%	Mean weight loss -4.2%, ITT: -2.1%			Not significant
2008	Brown A (15)	Exercise *vs*. no exercise	Median weight change -1.29%			Median weight change +0.45%	*P* < 0.001
2008	Thomson R (16)	Diet *vs*. diet + aerobic exercise *vs*. diet + aerobic resistance exercise	Mean weight loss -8.9 ± 1.6%	Mean weight loss -10.6 ± 1.7%	Mean weight loss -8.7 ± 1.7%		Not significant

### PCOS

PCOS is well acknowledged as a kind of disorder with both endocrine dysfunction and metabolic dysfunction (17), which impacts almost 20% of female patients of reproductive age. The symptoms of PCOS include androgen excess (hirsutism and/or hyperandrogenemia) and ovarian dysfunction (oligo-ovulation and/or polycystic ovarian morphology) (18). As studies focus on PCOS in-depth, insulin resistance and, subsequently, compensatory hyperinsulinism are regarded as two main mediators of hyperandrogenism in PCOS, both in animal models and in human bodies. It is widely acknowledged that hyperandrogenism and insulin resistance are common in PCOS. As an increasing number of studies focus on the phenotype and underlying mechanisms of PCOS, it is well demonstrated that insulin resistance and hyperinsulinism are responsible for the excessive androgen secretion in PCOS (19) since insulin could react on the ovary as a cogonadotropin and trigger androgen secretion from the adrenal glands. Therefore, it is suggested that insulin resistance and hyperinsulinism are the pathophysiological factors in the development of PCOS. Many studies hypothesized that PCOS might be triggered by hyperandrogenism together with some pathophysiological factors and subsequently promote insulin resistance and hyperinsulinism (20). Insulin resistance and hyperinsulinism could induce androgen secretion by the ovaries and adrenal glands in PCOS patients in return (21). Thus, a large proportion of women with androgen excess and/or ovulatory dysfunction are also disturbed by insulin resistance, suggesting that hyperandrogenism and insulin resistance are in a vicious circle in PCOS. One of the characterizations of PCOS is heterogeneity. In view of pathophysiological characteristics, the factors accounting for PCOS heterogeneity mainly include obesity, insulin resistance, abdominal adiposity, and so on. Studies have reported that peripheral insulin resistance (insulin resistance exists in muscle tissues and adipose tissues) is actually a characteristic of PCOS patients with obesity (18). Moreover, insulin resistance might be present even in PCOS women who are not obese (22). However, many studies have reported that hyperandrogenism might be one of the common characteristics (23). Hyperandrogenism alone would induce PCOS without any other factors as shown in some case reports (24). In some cases, PCOS would also be noticed to fully manifest with both hyperandrogenism and the above-mentioned pathophysiological characteristics (obesity, insulin resistance, and abdominal adiposity) (25). Thus, excess androgen might be necessary for developing PCOS. Considering that the frequent familial aggregation of PCOS might also be relative to the primary steroidogenic abnormality and the pathophysiological characteristics, it is not complicated to understand the genetic predisposition of PCOS. Recently, it is well acknowledged that familial aggregation is one of the characteristics in PCOS, which indicates that PCOS might be a kind of disorder with a certain genetic basis (26). However, enormous genome-wide relevant studies reported that the associations of only a few genetic variants and mutations have been replicated in different populations in PCOS patients (27). Familial aggregation might be linked to certain environmental influences which only exist in the families affected, including lifestyle during fetal and childhood development, certain environmental exposure and certain drugs, aging process as well as dietary habits, most of which might contribute to the development of PCOS (28). Based on the characteristic of familial aggregation of PCOS, it is reasonable to speculate that lifestyle modification might benefit the PCOS patients.

### Lifestyle Modifications

In recent years, lifestyle modifications are regarded as the cornerstone of all interventions against PCOS. Lifestyle modifications are regarded as the first-line management for patients disturbed by overweight or obesity (17). The most effective interventions include applicable diet modifications, increased physical activity and exercise modifications, and strategies to maintain adherence (29). Lifestyle modifications also appear to draw the ovulation function (30) as well as the menstrual cycle (31) into a regular level, which subsequently increases the successful pregnancy rates in PCOS patients. The studies reported that almost half of the PCOS patients would gain improvement both in regular menstrual cycle and ovulation function depending on the lifestyle modifications. In addition, lifestyle modifications could provide improvements such as alleviation of anxiety and improved quality of life, particularly in obese female patients with PCOS.

From the results shown in **Table 1**, a healthy eating diet and involvement in educational programs would lead to more weight loss—for example, Oberg E found that in terms of behavioral intervention, minimal intervention would help people attain weight loss (**Table 1**). The outcomes after diet modification, exercise modification, and the combination of diet with exercise are significantly different. A high-protein diet also leads to weight loss when compared with the standard protein diet. However, there was no significant difference between a vegan diet and a caloric diet. No significant difference was found between a low-GI diet and a hypocaloric healthy eating diet.

### Mechanism of Lifestyle Modifications in PCOS

Lifestyle modifications (including diet, exercise, sleep, and so on) are regarded to play roles in the development of PCOS by regulating insulin sensitivity and keeping the weight balanced as well as governing normal androgen production. It was reported that lifestyle changes also appear to influence the restoration of ovulation and regular menstrual cycles and increased the pregnancy rates in overweight or obese anovulatory patients with PCOS. It is widely acknowledged that obesity is a vital mediator in the development of PCOS. The level of sex-hormone-binding globulin is decreased in obese females (31), resulting in elevated androgen in the circulation and then in the target tissue, which disrupts normal ovulatory function (32). Additionally, obesity is associated with an elevated risk of metabolic syndrome, diabetes mellitus (type 2 diabetes), and insulin resistance in female bodies. Some studies compared the effects of lifestyle modifications with the effects of the combination of metformin and lifestyle modifications against PCOS and found that lifestyle modifications could reduce insulin resistance and increase the serum levels of sex-hormone-binding globulins when compared with metformin (33). Many studies also analyzed the effects of improved manifestations of PCOS by comparing the management of lifestyle modifications to the management of a combination of lifestyle modifications and other interventions (34). Negar reported their analysis based on 12 RCTs including 608 participants in which they witnessed a significant decrease in subcutaneous fat in subjects with “lifestyle (including daily physical activity, limited food intake, and so on) combined with metformin” compared with “lifestyle combined with placebo”. It was reported that both lifestyle modifications alone or a combination of lifestyle modifications and hormonal contraceptives have the potential to improve sexual function (35).

### Exercise Modifications

As increasing studies focus on the roles of physical activities in human health, the evidence showed that in the management of PCOS, exercise activities would help female patients gain benefits, and this view is becoming accepted among doctors and patients (36, 37). When considering the appropriate exercise activities to alleviate the symptom of PCOS, it is always puzzling how to set the appropriate exercise intensity and frequency. Recently, a meta-analysis reported that improvements in health outcomes are more likely to be linked to the exercise intensity rather than the exercise itself. An RCT study indicated exercise modifications with vigorous intensity (eight consecutive weeks and three sessions of supervised exercise training each week for the final four consecutive weeks). Each session lasts approximately 60 min and will involve 40 min of an individualized exercise protocol performed either on a cycle ergometer or a motorized treadmill preceded by a 10-min warm-up and followed by a 10-min cool-down) might have a better impact on the outcomes of PCOS (insulin resistance decreased significantly) (38). On the contrary, PCOS patients are found to be more likely to stay sedentary rather than perform vigorous exercises. Moderate aerobic exercise could also improve the insulin sensitivity of PCOS in the short term. Some other studies reported that women with PCOS could gain improvement, in terms of insulin sensitivity and abnormal androgen level, *via* vigorous aerobic exercise and resistance training (39). The minimum aerobic activity is recommended as more than 150 min per week, including intensive exercise for more than 90 min (2).

### Diet Modifications

While it is recommended to reduce the calorie intake and induce weight loss among PCOS women with obesity, most of the current proposed recommendations regarding dietary modifications in PCOS are based on studies in obese women without PCOS. It was reported that there is limited evidence that any specific diet type is better than others (40). Some studies reported that once the intake of carbohydrates is less than 45% of the total daily calories, the low-carbohydrate diet might be helpful to decrease the body mass index as well as the serum levels of total cholesterol in PCOS subjects (41). Furthermore, studies indicate that maintaining the low-carbohydrate diet for more than 1 month could significantly increase the levels of follicle-stimulating hormone and sex-hormone-binding globulin (36). Even though some evidence indicates the effect of the low-carbohydrate diet on PCOS, the definitive mechanisms to explain the relationship are still unclear. It is well acknowledged that metformin has similar effects in decreasing body weight. Some studies compared the effects of diet modifications with the effects of the combination of metformin and lifestyle modifications against PCOS. It was reported that diet modifications could reduce insulin resistance and increase the serum levels of sex-hormone-binding globulins when compared with metformin (34).

What is more, weight loss could improve the features of PCOS patients regardless of dietary composition (42). Unfortunately, lifestyle modifications, including diet modifications, are seldom effective in the long run, which are in line with the results from the management of anti-obesity drugs. The unsatisfactory long-term results might be associated with the fact that the female subjects regain weight and fail to keep a normal body mass index (BMI).

### Weight Modifications

While the complex clinical heterogeneity of PCOS brings up the lack of a clear understanding of obesity in PCOS, it is widely accepted that obesity would increase insulin resistance and hyperandrogenism. It was reported that just a minor weight loss of 5–10% could play a role in significantly alleviating reproductive disorders (43), metabolic dysfunction, and even the psychological symptoms of PCOS patients (44). Thus, weight modification is recommended as a first step in the management of PCOS patients who are overweight or obese (45).

If the PCOS patient is disturbed by infertility, it is recommended that women with PCOS and obesity should delay therapy against infertility and achieve the weight modifications first because obesity is linked to a higher risk of increased rates of miscarriage and preeclampsia in perinatal PCOS women. It is believed that PCOS patients who are overweight/obese are more likely to face mood disorders, including anxiety, depression, and so on. However, the degree of the increased risk of excess weight and the impact on the prevalence and severity of the features of PCOS remains unclear. Anti-obesity drugs, including orlistat, could also be considered in PCOS patients who cannot achieve weight modification with diet modification and exercise modification (45, 46). Women with PCOS and normal weight and BMI also have an increased risk for metabolic disorders and chronic fatigue. A similar exercise program combined with diet modification is also recommended because these modifications could enhance insulin sensitivity (47, 48). Moreover, more studies are needed on the effects of weight modifications on normal-weight patients with PCOS. Thus, it is recommended that females with PCOS pursue weight modifications and prevent excessive weight gain by weight monitoring and maintaining appropriate BMI and waist circumference.

### Mood Modifications

An increasing volume of evidence shows that both adolescent and adult females with PCOS are disturbed by mood disturbances, including depression and anxiety (49). It was reported that females with PCOS underwent a higher risk of depression, anxiety, and perceived stress when compared with women without PCOS (50). Since PCOS is linked to an increased risk of depression, anxiety, and some other mood disorders, screening and effective mood modifications for these disorders might be warranted.

There are several shared links and connections between depression- and PCOS-associated abnormalities, such as excessive androgen secretion, insulin resistance as well as obesity. These shared connections between depression and PCOS might help in finding potential therapies for depression in PCOS (51). A kind of appropriate and applicable mood modification for the two treatments is regarded as the potential modification which could help females with PCOS to lead a better life.

### Sleep Modifications

It is important that psychological issues are considered as both a potential risk and a maintaining factor of illness, particularly in adolescent and young female subjects (52). As per the in-depth investigation and data analysis, a large proportion of psychological disorders with PCOS are sleep disorders. Since sleep disorders impact the development of PCOS, management relative to sleep modifications is considered an integral part of lifestyle modifications on females with PCOS. There is ample evidence that sleep deprivation is associated with an increased risk of insulin resistance and obesity as well as type 2 diabetes (53). The mechanisms of the associations have been proven to be linked to relative autonomic pathways, endocrine disorders, and inflammatory status, which are responsible for the development of PCOS (54). Therefore, it is plausible that sleep modifications are of great significance among PCOS patients. Some studies reported that women are more likely to be disturbed by type 2 diabetes if the length of sleep is not more than 5 h per night when compared with women whose length of sleep ranges from 7 to 8 h per night. A study compared the quality of sleep by recording the percentage of rapid eye movement sleep *via* polysomnography and found that the percent sleep efficiency of obese females with PCOS is lower than that of not only normal-weight females but also obese adolescent females without PCOS (55). Ensuring adequate sleep with high quality would lead to a decreased risk of disturbance not only in obesity and insulin resistance but also in cardiovascular risk, suggesting that sleep modification could modify PCOS as an original modification.

## Summary and Perspectives

PCOS is a kind of common endocrine and metabolic disorder which disturbs female subjects at reproductive ages. Since increasing evidence indicates that PCOS is frequently linked to abdominal adiposity, insulin resistance, obesity, metabolic disorders, and cardiovascular risk factors and becomes a complex disorder with environmental effects, such as diet and other lifestyle factors, lifestyle modification is therefore regarded as the first line of management for PCOS patients.

PCOS women who are overweight or obese not only are exposed to metabolic and cardiovascular risk but also suffer from potential mental problems and risk adverse obstetric and perinatal outcomes, such as gestational diabetes and preterm birth. Obesity leads to high healthcare costs, which means that it would increase the investment in lifestyle modification. In **Table 1**, our data suggested that a healthy diet and increased physical activity should be encouraged for weight loss, which was in line with many published suggestions or guidelines. The conclusion would undoubtedly propose new insights and promising strategies to support clinical practices.

We review the lifestyle modifications in PCOS, including diet modifications, exercise modifications, sleep modifications, mood modifications, and weight modifications. While physical modification, appropriate dietary modification, and maintaining healthy sleep modification and mood modification are recommended for the management of various PCOS conditions, more perspective studies are needed on the effects of lifestyle modifications on PCOS to figure out and develop accurate and individualized guidelines. Lifestyle modifications in PCOS are not old stories but also new tales.

## Author Contributions

YG contributed to writing—original draft. GZ contributed to writing—original draft and editing. FZ contributed to writing—review and editing. QW, CM, and JD contributed to review and editing. KH contributed to writing—review and editing and supervision. All authors contributed to the article and approved the submitted version.

## Conflict of Interest

The authors declare that the research was conducted in the absence of any commercial or financial relationships that could be construed as a potential conflict of interest.

## Publisher’s Note

All claims expressed in this article are solely those of the authors and do not necessarily represent those of their affiliated organizations, or those of the publisher, the editors and the reviewers. Any product that may be evaluated in this article, or claim that may be made by its manufacturer, is not guaranteed or endorsed by the publisher.
